# Overexpression of the Notch3 receptor and its ligand Jagged1 in human clinically non-functioning pituitary adenomas

**DOI:** 10.3892/ol.2013.1113

**Published:** 2013-01-07

**Authors:** RUNCHUN LU, HUA GAO, HONGYUN WANG, LEI CAO, JIWEI BAI, YAZHUO ZHANG

**Affiliations:** 1 Beijing Neurosurgical Institute, Beijing Tiantan Hospital, Capital Medical University, Dongcheng, Beijing 100050, P.R. China;; 2 Neurosurgical Department, Beijing Tiantan Hospital, Capital Medical University, Dongcheng, Beijing 100050, P.R. China

**Keywords:** Notch signal pathway, Notch3, Jagged1, γ-secretase inhibitor, human clinically non-functioning pituitary adenoma, pathogenesis

## Abstract

Human clinically non-functioning pituitary adenomas (NFPAs) primarily cause headaches, visual impairment and hypopituitarism due to the effect of the mass of the tumor. Surgery is the first-line treatment for these tumors. To date, no efficacious medical therapy exists for non-functioning adenomas. Previous studies have demonstrated that the Notch3 receptor is involved in the pathogenesis of various types of malignancies, including human NFPAs. The current study focused on the expression of the Notch3 receptor and its ligand Jagged1 in three types of pituitary adenomas and in the normal pituitary gland. Using quantitative real-time RT-PCR assays and western blot analyses, upregulated Notch3 and Jagged1 were observed in human NFPAs, but not in normal human pituitary glands or in hormone-secreting adenomas. Furthermore, Notch3 was positively correlated with Jagged1 at the mRNA and protein levels. These data indicate that Notch3 and Jagged1 may play an important role in the initiation and proliferation of human non-functioning adenomas, and there may be an interaction between Notch3 and Jagged1 in this process. Our study further elucidates the role of the Notch3 signaling pathway in the tumorigenesis of human NFPAs and provides a potential therapeutic target for the medical treatment of these tumors.

## Introduction

Human clinically non-functioning pituitary adenomas (NFPAs) are defined as adenomas lacking symptoms or signs secondary to oversecretion of pituitary hormones by the tumor. Non-functioning adenomas constitute 9–50% of pituitary tumors and ∼80% of pituitary macroadenomas (1,2). The majority of patients with NFPAs seek medical attention due to the mass effects of the tumor, which include headaches, visual impairment and hypopituitarism (3,4). However, ∼9% of NFPAs are subclinical adenomas which present as an incidentally found mass in the sellar region (3). The majority of NFPAs are not accompanied by hypersecretion of significant pituitary hormones in serum, with the exception of mild hyperprolactinemia in certain cases (5). However, by immunocytochemistry, the large majority of such tumors synthesize pituitary glycoprotein hormones (follicle-stimulating hormone, FSH; luteinizing hormone, LH; thyroid-stimulating hormone, TSH; growth hormone, GH and prolactin, PRL) and/or their free subunits (α-subunit, β-FSH, β-LH and β-TSH) (4,6,7). Pathologically, NFPAs are classified into gonadotroph, silent and null cell adenomas, in which no immunoreactive hormone or its corresponding subunit is found (8). For NFPAs, surgery is the first-line treatment. Radiotherapy should be considered for residual tumors, while asymptomatic patients should be followed up conservatively (3,4). Effective medical therapy has been demonstrated in functioning pituitary adenomas (9). Medication, such as dopamine agonists and somatostatin analogs, has been prescribed for NFPAs; however, the efficacy has not been satisfactory, with the exception of a small number of case studies (9,10). Thus, there is a need for specific and efficacious medical treatments for patients bearing NFPAs.

Notch signaling is a highly evolutionarily conserved pathway involved in various functions during development, including cell fate control, the maintenance of stem cells and apoptosis (11–13). The basic components of this pathway are Notch receptors, ligands and transcription factors. In humans, there are four Notch receptors (Notch1, 2, 3 and 4), two Jagged ligands (Jagged1 and Jagged2) and three δ–like ligands (Dll1, Dll3 and Dll4) (14). The activation of Notch by its ligand frees the intracellular domain of Notch (Notch-IC) and enables it to enter the nucleus through a cascade of proteolytic cleavages by α- and γ-secretase. In the nucleus, Notch-IC then activates the transcription of Notch target genes, primarily by binding to a ubiquitous transcription factor, CSL. The CSL pathway includes CBF-1 (also known as RBP-Jκ), Suppressor of Hairless [Su(H)] and Lag-1 (12,15). In addition to their function in developmental processes, increasing evidence demonstrates that Notch-ligand interactions also participate in the pathogenesis of a number of human diseases. Differential expression of the Notch3 protein and its ligand Jagged1 has been demonstrated in numerous human malignancies. Activating mutations of Notch3 are present in human T-cell acute lymphoblastic leukemia and a number of human solid tumors, including ovarian, breast and colorectal cancer (16–19). Additionally, Jagged1 is involved in the progression and proliferation of various human tumors through its interaction with the Notch3 receptor in human ovarian carcinoma, multiple myeloma and colorectal cancer (20–23). These observations suggest that the binding of Jagged1 to Notch3 contributes to the onset and progression of human tumors by activation of the Notch signaling pathway. Two microarray gene expression studies conducted by Moreno *et al* revealed that *Notch3* was overexpressed in human NFPAs and PRL-secreting pituitary adenomas (24,25). Another study demonstrated elevated Notch3 mRNA and protein expression in non-functioning pituitary tumors (26). However, the role of Jagged1 in pituitary adenomas has not yet been demonstrated, and there is no direct evidence of the function of Notch3 in GH- and PRL-secreting adenomas. In the current study, we investigated the role of Notch3 and its ligand Jagged1 in various types of pituitary adenoma as well as in normal pituitary glands. We provide the first description of the differential expression of Jagged1 in human pituitary adenomas and its correlation with Notch3.

## Materials and methods

### Patients and tissues

Seventeen pituitary adenomas were obtained from patients at the Beijing Tiantan Hospital during endoscopic transsphenoidal surgery, and three normal human adenohypophyses were obtained from a donation program. Informed consent was obtained from the patients and the study was approved by the Ethics Committee of Beijing Tiantan Hospital, Beijing, China. All samples were rinsed in sterile saline, snap-frozen in liquid nitrogen and then stored in liquid nitrogen until analysis. Clinical details of the patients are summarized in Table I. Individual adenomas were classified based on the profile of adenohypophyseal hormone content, by histology and immunohistochemistry prior to molecular analysis.

### RNA extraction and quantitative real-time reverse transcription-polymerase chain reaction (RT-PCR) assay

Total RNA was extracted from frozen pituitary adenomas and normal pituitaries (40–60 mg) using TRIzol reagent (Invitrogen Life Technologies, Carlsbad, CA, USA) and first-strand cDNA was synthesized from total RNA using the SuperScript First-Strand Synthesis system with SuperScript II reverse transcriptase, according to the manufacturer’s instructions (Invitrogen Life Technologies). RT-PCR was performed in an Applied Biosystems 7500 Fast system using Platinum SYBR-Green/ROX qPCR Supermix-UDG (Invitrogen Life Technologies). The qPCR reaction system was performed in a 25-*μ*l reaction, which comprised 2X Master mix (12.5 *μ*l), forward/reverse primers (0.5 *μ*l each, 10 *μ*mol/l), sample cDNA (1 *μ*l) and double distilled water (ddH_2_O; 10.5 *μ*l). The amplification conditions were 50°C for 120 sec, 95°C for 120 sec, as well as 40 cycles at 95°C for 15 sec and 60°C for 30 sec. The fluorescence of the PCR products was read following completion of the extension step. The expression of mRNA was determined from the threshold cycle (CT), and the relative expression levels of the tested genes were normalized relative to that of *GAPDH* and calculated from the CT value using the 2^−ΔΔCT^ method for quantification (27). The primers used in the RT-PCR assay are listed in Table II.

### Protein preparation and western blot analysis

Pituitary adenomas or normal pituitary gland tissue from humans were homogenized in lysis buffer in a handheld micro-tissue homogenizer. The homogenate was then centrifuged at 12,000 × g for 15 min at 4°C, and the supernatant was denatured for 5 min at 95°C in loading buffer. Protein concentrations were measured using the bicinchoninic acid protein assay with bovine serum albumin as the standard. Soluble proteins (60 *μ*g) were separated by electrophoresis in 8 or 10% sodium dodecyl sulfate polyacrylamide gels, transferred to nitrocellulose membranes and incubated with blocking buffer (5% non-fat milk in Tris-buffered saline Tween-20 (TBST) for 1 h at room temperature. Membranes were then probed overnight with the corresponding primary antibody at 4°C, followed by three 10 min washes with TBST. Subsequently, membranes were incubated with secondary antibodies conjugated with horseradish peroxidase at room temperature for 1 hour. Rat polyclonal Notch3 antibody (1:1,000, 8G5; Cell Signaling Technology, Inc., Boston, MA, USA) and rabbit polyclonal Jagged1 antibody (1:1,000, 28H8; Cell Signaling Technology, Inc.) were used. Enhanced chemiluminescence, performed according to the manufacturer’s instructions (Amersham Pharmacia Biotech, Piscataway, NJ, USA) was used to demonstrate positive bands that were visualized after exposure on a transparent medical X-ray film. The final data were subjected to grayscale scanning and semi-quantitative analysis using Quantity One software (Bio-Rad, Hercules, CA, USA).

### Statistical analysis

All data are expressed as the mean ± standard error. Statistical analyses of protein expression between tumor types were performed using Student’s t-tests or non-parametric Mann-Whitney U tests. Correlations were performed using the Pearson Rank Sum test. P<0.05 was considered to indicate a statistically significant difference. The Statistical Package for the Social Sciences version 17.0 (SPSS; SPSS Inc., Chicago, IL, USA) was used for statistical analyses.

## Results

### Tumor classification

The clinical and pathological characteristics of the 17 adenomas used in this study are listed in Table I. There were nine male and eight female patients. The average age of the patients was 41 years (range, 22–55) and the average tumor diameter was 3.3 cm (range, 1.7–7.1). There were eight NFPAs, five GH-secreting adenomas and four PRL-secreting adenomas. Four NFPAs were not positive with anterior pituitary hormone histochemistry and were designated immunohistochemically negative (NF^−^) tumors, while four NFPA tumors were stained with LH and/or FSH and were designated immunohistochemically positive (NF^+^). The four PRL-secreting adenomas manifested as hyperprolactinemia, while the five GH-secreting adenomas were characterized with acromegaly. For the eight NFPAs, headache and visual defects were the main symptoms. Three normal pituitary controls were obtained from a donation program, and these patients did not have any endocrinological diseases.

### RT-PCR analysis

**Figure 1:**
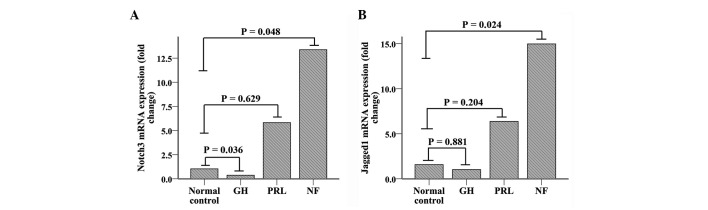
RT-PCR analysis results. (A) RT-qPCR of relative expression of *Notch3* mRNA in NFPAs (n=6), GH-secreting (n=5) and PRL-secreting adenomas (n=4), compared with normal pituitary tissue (n=3). Increased expression of *Notch3* is demonstrated in NFPAs (P<0.05). Decreased *Notch3* expresion is also evident in GH-secreting adenomas (P<0.05). (B) RT-qPCR of relative expression of *Jagged1* mRNAs in NFPAs (n=6), GH-secreting (n=5) and PRL-secreting adenomas (n=4), compared with normal pituitary tissue (n=3). Elevated expression of *Jagged1* is only demonstrated in NFPAs (P<0.05).

*Notch3* mRNA expression (Fig. 1A) increased ∼6.5-fold in NFPAs (n=6), compared with normal pituitary tissue controls (n=3, P=0.048). Although PRL-secreting adenomas (n=4) demonstrated a 1.5-fold increase in *Notch3* mRNA compared with normal pituitary tissue, there was no significant difference between these two groups (P=0.629). GH-secreting adenomas (n=5) demonstrated significantly reduced expression (∼75% reduction) of *Notch3* compared with normal tissue (P=0.036). Overall, pituitary adenomas (n=15) demonstrated a 4-fold increase in *Notch3* mRNA expression compared with normal pituitary tissue; however, this increase was not significantly different (P=0.100). Additionally, nonfunctioning adenomas demonstrated increased expression of *Notch3* compared with functioning adenomas, which included PRL- and GH-secreting adenomas (P=0.026).

*Jagged1* mRNA expression (Fig. 1B) was also markedly increased (∼11.2-fold) in NFPAs (n=6) compared with normal pituitary tissues (n=3, P=0.024). PRL-secreting adenomas (n=4) demonstrated a 3.9-fold increase in *Jagged1* mRNA expression compared with normal pituitary tissue, although the difference was not statistically significant (P=0.204). In contrast to its receptor (Notch3), *Jagged1* mRNA expression levels in GH-secreting adenomas were similar to normal (P= 0.881). There was no significant difference between all pituitary adenomas and normal pituitary tissue (P=0.824). As with its receptor, *Jagged1* mRNA expression significantly increased in NFPAs compared with functioning adenomas (P=0.005).

### Western blot analysis

**Figure 2:**
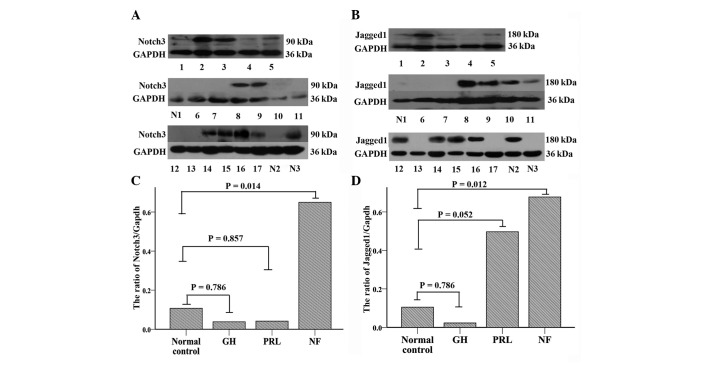
Western blot analysis. (A and B) Expression of Notch3 and Jagged1 protein in NFPAs (n=8, samples 2, 3, 8, 9, 14, 15, 16 and 17), GH-secreting adenomas (n=5, samples 4, 5, 6, 7 and 13), PRL-secreting adenomas (n=4, samples 1, 10, 11, 12 and 13) and normal pituitary tissue (n=3, samples N1, N2 and N3). GAPDH was used for normalization. (C and D) Western blot analysis reveals a significant increase in the Notch3 and Jagged1 proteins in NFPAs compared with the normal pituitary tissue (P<0.05).

Western blot analysis (Fig. 2A and C) demonstrated that Notch3 protein expression was consistent with the mRNA findings and was significantly increased in NFPAs (n=8) compared with normal pituitary tissue (n=3, P= 0.014). Notch3 protein expression levels of GH- and PRL-secreting adenomas were similar to those of normal pituitary tissue (n=5, P>0.05; n=4, P>0.05, respectively). As a group, pituitary adenomas (n=17) did not demonstrate an elevated expression of Notch3 protein compared with normal pituitary tissue (n=3, P=0.335). Unlike RT-PCR analysis, Notch3 protein expression was significantly elevated in NFPAs compared with functioning adenomas (n=9, with 5 GH-secreting adenomas and 4 PRL-secreting adenomas; P=0.002).

Consistent with the RT-PCR findings, Jagged1 protein expression (Fig. 2B and D) in NFPAs (n=8) was elevated significantly compared with that in normal pituitary glands (n=3, P=0.012). As for GH-secreting (n=5) or PRL-secreting adenomas (n=4), the Jagged1 expression levels were not significantly different from those in normal tissue (P=0.786 and P=0.052, respectively). Additionally, pituitary adenomas (n=17) and normal pituitary glands (n=3, P=0.18) expressed similar Jagged1 levels. Although the protein expression level of Jagged1 was not statistically significantly different between NFPAs and functioning adenomas (P>0.05), NFPAs exhibited a significantly increased expression of Jagged1 compared with GH-secreting adenomas (P=0.011).

### Correlation between expression of Notch3 and Jagged1

**Figure 3:**
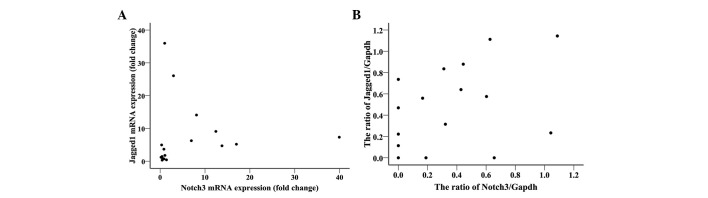
Scatter diagrams demonstrating the correlation between Notch3 and Jagged1 expression. (A) RT-qPCR analysis reveals that the expression of the Notch3 transcript is positively correlated with Jagged1 expression (Pearson’s correlation coefficient = 0.560, n=20, P=0.016). (B) Western blot analysis demonstrates a similar correlation between the corresponding proteins (Pearson’s correlation coefficient = 0.532, n=8, P=0.012).

The expression of Notch3 mRNA was positively correlated with *Jagged1* mRNA expression (Fig. 3A), with a Pearson’s correlation coefficient of 0.560 (n=20, P=0.016). Similarly, a positive correlation was observed between the expression of *Notch3* and Jagged1 proteins (Fig. 3B), with a Pearson’s correlation coefficient of 0.532 (n=18, P= 0.012).

## Discussion

As significant oncogenes in humans, Notch receptors and their ligands have been demonstrated to be involved in the pathogenesis of numerous neoplasms via various mechanisms. Notch3 affects apoptosis and tumor growth in lung cancer by co-operating with the EGFR-MAPK pathway (28,29). Additionally, Notch3 promotes proliferation and inhibits apoptosis of ErbB2-negative breast tumor cells via activation of the CSL (CBF-1/RBP-Jκ, Su(H) and Lag-1) pathway (18). Also, through the canonical CSL-mediated transcriptional network, Notch3 is capable of regulating esophageal squamous cell differentiation and proliferation (30). In addition to solid tumors, Notch3 is able to promote the survival of T acute lymphoblastic leukemia cells via regulation of MKP-1, which is a member of the MAPK pathway (31). However, the regulatory functions of Notch3 described previously require activation by Notch3 ligands. Abundant evidence suggests that the interaction between Notch3 and Jagged1 plays a key role in the tumorigenesis of numerous diverse malignancies, including ovarian cancer, colorectal cancer and multiple myeloma cells (20–22). Thus, Jagged1 is an important ligand of Notch3 and is involved in the pathogenesis of neoplasms.

In the present study, we provide the first description of the differential expression of the *Notch3* receptor and its ligand Jagged1 in various types of human pituitary adenomas at the mRNA and protein levels. The expression of Notch3 mRNA and protein was significantly elevated in the NFPAs compared with normal pituitary tissue, whereas all pituitary adenomas do not overexpress Notch3. Our results are consistent with the results of previous studies. Overexpression of Notch3 had been observed in human clinically NFPAs in the study by Miao *et al*(26). In this study, additional immunohistochemical analyses were performed, demonstrating that the Notch3 receptor is primarily expressed in the cytoplasm of NFPAs. Gene microarrays and proteomic analyses have demonstrated that Notch3 gene and protein expression are increased in human clinically NFPAs (24,32). However, in the study conducted by Moreno *et al*(24), all types of non-functioning adenomas were evaluated, whereas in our study, only two types (NF^−^, LH/FSH^+^) were analyzed. These results suggest that Notch3 may play a significant role in the development of human NFPAs other than GH- and PRL-secreting adenomas. However the exact mechanism of Notch3 in the tumorigenesis of NFPAs remains to be elucidated.

In the current study, we have provided evidence of Notch3 expression in human functioning adenomas, a finding that has not been previously reported. The expression of Notch3 was moderately elevated in NFPAs compared with functional adenomas, which included GH- and PRL-secreting adenomas. However, ACTH- and TSH-secreting adenomas were not evaluated in our study. The function of Notch in the development and cell specification of the pituitary gland has been explained by several studies (33–35). Activation of Notch in zebrafish has been reported to lead to the loss of lactotropic cell specification, and increase the number of gonadotropes, corticotropes and melanotropes in the anterior pituitary (35). Another study demonstrated that Notch regulates the specification of diverse cell types in the pituitary of mice (34). Notch has been demonstrated to repress Math3, which is a Pit1 target gene that is specifically required for the maturation and proliferation of the GH-producing somatotrope (34). Coincidently, our data demonstrated that Notch3 receptor expression was slightly decreased in GH-secreting adenomas compared with controls, although those changes were not statistically significant. The aforementioned studies, together with our experimental data, imply that Notch may regulate the specification of cell types in pituitary adenomas. In addition, Notch3 may promote the maturation and proliferation of gonadotropes, which are the predominant cell type of NFPAs. GeneChip microarrays and proteomic analyses have demonstrated an increased expression of Notch3 in PRL-secreting adenomas (25). Our data demonstrated paralleled expression of Notch3 between PRL-secreting adenomas and normal controls. Notably, the adenomas used in the previous study were larger in size (diameters were >3 cm) and patients presented with markedly elevated serum prolactin levels (>1000 ng/ml), compared with those in our study. These differences imply that Notch3 may stimulate the growth and hormone production of PRL-secreting adenomas, although the molecular mechanism involved requires clarification.

Ligands of the Notch3 receptor also play a role in the pathogenesis of pituitary adenomas. Previous studies have demonstrated that Dll1, a potential ligand of Notch3, was strongly downregulated in non-functional tumors and in PRL-secreting adenomas (24,25). In the present study, we have provided the first evidence of Jagged1 overexpression in NFPAs. Our data demonstrated that Jagged1 expression, similar to the Notch3 receptor but opposite to Dll1, was increased in NFPAs compared with the control pituitary tissue. A positive correlation was also observed between Notch3 and Jagged1 expression at the mRNA and protein levels. These results imply that there is a link between Jagged1 and Notch3 in the pathogenesis of NFPAs. Abundant evidence describes the participation of Jagged1 in the angiogenesis and tumorigenesis of various types of malignancies, including ovarian and colorectal cancer, squamous cell carcinoma and multiple myeloma (20–23,36,37). The majority of these studies revealed that the interaction between Notch3 and Jagged1 participates in the growth, proliferation and angiogenesis of the tumors (20–23,36). The present study also demonstrated that Jagged1, like the Notch3 receptor, has a higher expression level in NFPAs than in functioning adenomas. Thus, we speculate that Jagged1 may play an important role in the specification, initiation and/or proliferation of NFPAs via the activation of the Notch3 pathway. However, the exact mechanism of the interaction between Notch3 and Jagged1 in pituitary adenomas remains to be elucidated.

The Notch signaling pathway regulates the initiation, specification and proliferation of neoplasms, primarily through two different mechanisms. The first mechanism is the canonical CSL-mediated transcriptional network. Ligand binding leads to the release of the Notch-IC, which then translocates into the nucleus and interacts with the CSL DNA-binding protein to generate a transcriptional activator complex. The latter induces expression of target genes, including Hes/Hey family genes, cyclin D and NF-κB. These genes participate in cell specification, growth, progression and survival (11).

Notch3-induced activation of NF-κB induces differentiation or neoplastic transformation of T-cells (38,39). *Notch3* promotes tumor cell growth and proliferation via the Hes gene in a CSL-dependent fashion (22,30). The Notch3 and Hes genes regulate the differentiation and specification of progenitor cells in pituitary development (40,41). Cyclin D1, one of the Notch target genes, has been demonstrated to be overexpressed in NFPAs (42). These data suggest that the Notch signaling pathway may regulate the pathogenesis of NFPAs via the canonical CSL-mediated transcriptional network. Until now, there was no direct evidence to support this hypothesis. The second mechanism of the Notch3 promotion of tumorigenesis is through co-operation with other signaling pathways. Interaction between Notch3 and other signaling pathways, including the Wnt, MAPK, and EFGR pathways, plays a key role in the growth and proliferation of various neoplasms (21,28,36). Further study is required to elucidate the exact mechanism of Notch3 signaling in pituitary tumor development and tumorigenesis.

As the Notch signaling pathway is involved in the pathogenesis of numerous diverse malignancies, it is plausible that inhibition of this pathway may have antitumor effects. Following activation of the Notch receptor, the proteolytic processing of Notch by the γ-secretase protein complex is an essential step that leads to the release of the Notch-IC and transcription of target genes (43). Therefore, γ-secretase inhibitors are capable of specially blocking Notch signaling pathway activation. It has been demonstrated that γ-secretase inhibitors suppress proliferation and induce apoptosis in T-cell leukemia, and in lung and breast cancer (44–46). Furthermore, γ-secretase inhibitors have been used to treat lymphoma in pre-clinical trials (47). However, the anti-oncogenic potential of γ-secretase inhibitors in NFPAs requires further study.

In conclusion, the present study demonstrated increased Notch3 and Jagged1 expression, as well as a positive correlation between Notch3 and Jagged1, in human NFPAs. Further studies are required to elucidate the exact mechanism whereby the Notch signaling pathway participates in the pathogenesis of NFPAs. The present study implies that the Notch signaling pathway may be a potential therapeutic target in the treatment of NFPAs.

## Figures and Tables

**Table I:** Clinical and pathological characteristics of the 17 pituitary adenomas from the patients in this study.

Patient ID	Gender	Age (years)	Tumor size (cm)	Clinical characteristics	Immunohistochemical analysis
1	M	32	2.4	Hyperprolactinemia	PRL^+^
2	F	43	3.5	Headache and visual defects	NF^−^
3	M	55	3.0	Visual defects	NF^+^: FSH^+^
4	F	48	2.2	Acromegaly	GH^+^
5	M	33	1.7	Acromegaly	GH^+^
6	M	52	3.6	Acromegaly	GH^+^
7	F	41	4.9	Acromegaly	GH^+^
8	F	55	2.8	Symptomless	NF^+^: LH^+^, FSH^+^
9	M	51	4.5	Headache and hypopituitarism	NF^+^: LH^+^, FSH^+^
10	M	22	4.4	Hyperprolactinemia	PRL^+^
11	F	35	2.0	Hyperprolactinemia	PRL^+^
12	F	46	2.5	Hyperprolactinemia	PRL^+^
13	F	26	1.8	Acromegaly	GH^+^
14	M	46	4.6	Headache and visual defects	NF^−^
15	F	37	7.1	Headache, visual loss and hydrocephalus	NF^−^
16	M	47	2.6	Headache	NF^+^:LH^+^
17	M	29	3.0	Visual defects	NF^−^

PRL, prolactin; NF, non-functioning pituitary adenomas; FSH, follicle-stimulating hormone; GH, growth hormone; LH, luteinizing hormone.

**Table II:** Primers used for RT-PCR in this study.

Gene name	Amplification (bp)	Forward sequence (5′ to 3′)	Reverse sequence (5′ to 3′)	Temp. (°C)
*Notch3*	141	TGGCGACCTCACTTACGACT	CACTGGCAGTTATAGGTGTTGAC	60.9
*Jagged1*	77	GGGGCAACACCTTCAACCTC	CCAGGCGAAACTGAAAGGC	60.0
*GAPDH*	228	GAAGGTCGGAGTCAACGGATT	CGCTCCTGGAAGATGGTGAT	60.0

RT-PCR, reverse transcription-polymerase chain reaction.
